# Plasma Interleukin-37 Is Elevated in Patients with Rheumatoid Arthritis: Its Correlation with Disease Activity and Th1/Th2/Th17-Related Cytokines

**DOI:** 10.1155/2015/795043

**Published:** 2015-09-06

**Authors:** Ting Xia, Xing-feng Zheng, Bao-hua Qian, He Fang, Jun-jie Wang, Lan-ling Zhang, Ya-fei Pang, Ju Zhang, Xiao-qing Wei, Zhao-fan Xia, Dong-bao Zhao

**Affiliations:** ^1^Department of Rheumatology, Changhai Hospital, Second Military Medical University, Shanghai 200433, China; ^2^Department of Burn Surgery, Changhai Hospital, Second Military Medical University, Shanghai 200433, China; ^3^Department of Blood Transfusion, Changhai Hospital, Second Military Medical University, Shanghai 200433, China; ^4^Tissue Engineering and Reparative Dentistry, School of Dentistry, Cardiff University, Heath Park, Cardiff CF14 4XY, UK

## Abstract

Interleukin- (IL-) 37 is a novel anti-inflammatory cytokine that suppresses immune response and inflammation. This study was performed to determine whether IL-37 was elevated in patients with rheumatoid arthritis (RA) and investigate the correlation between IL-37 level and disease activity and the concentration of Th1/Th2/Th17-related cytokines. Clinical parameters of disease activity, including the 28-joint disease activity score (DAS28) and C-reactive protein (CRP), were collected in 34 RA patients and 34 age- and sex-matched healthy controls. Plasma IL-37 was measured by ELISA. Plasma levels of TNF-*α*, IL-1*β*, IL-2, IL-4, IL-5, IL-6, IL-7, IL-8, IL-10, IL-12, IL-13, IL-17, G-CSF, GM-CSF, IFN-*γ*, MCP-1, and MIP-1*β* were analyzed using the Bio-Plex suspension array system. It was found that IL-37 levels were elevated markedly in RA patients and almost undetectable in healthy controls. In addition, IL-37 levels in patients with active RA were significantly enhanced as compared with those in patients of remission. More importantly, IL-37 showed a significant correlation with disease activity (DAS28) and IL-4, IL-7, IL-10, IL-12, and IL-13 concentrations in RA patients. These findings suggest that IL-37 plays an important role in the pathogenesis of RA and may prove to be a potential biomarker of active RA.

## 1. Introduction

Rheumatoid arthritis (RA) is a systemic autoimmune inflammatory disorder primarily affecting the peripheral joints. The exact pathogenesis of RA remains elusive, though strong evidence suggests the involvement of cytokines in the disease progression. It is clear that cytokines play a fundamental role in various inflammatory processes, articular destruction, and RA-associated comorbidities [1]. Single-cytokine target therapies such as TNF blockade have proved useful for the treatment of RA patients [2]. However, nonresponders or partial clinical responders are not infrequently seen and disease often flares up upon discontinuation of treatment. Thus, considerable unmet clinical needs remain, and biology and relevant pathophysiology of novel cytokines in RA need to be further explored.

Recent studies have shown that IL-37 is a key cytokine in regulating inflammatory response, mainly by inhibiting the expression, production, and function of proinflammatory cytokines. IL-37, formerly named IL-1F7, shares similar structural pattern to the IL-1 family. IL-37 is widely expressed in several types of cells, organs, and tissues, including monocytes, plasma cells, dendritic cells, the testis, thymus, and uterus. IL-37 is believed to play potential roles in autoimmune diseases by suppressing immune responses and inflammation. Immunohistochemistry staining on the synovial tissue from individuals with active RA demonstrated the presence of large amounts of IL-37 in the diseased synovial lining [3].

Numerous cytokines, both proinflammatory and anti-inflammatory, have been detected in RA patients, and the balance between the activities of these opposing cytokines determines disease severity [4]. An imbalance between pro- and anti-inflammatory cytokine activities favors the induction of autoimmunity, chronic inflammation, and thereby joint damage [5]. Given the correlation between cytokines and disease symptoms of RA patients, some Th1/Th2/Th17-related cytokines or multicytokine profiles were detected as the important biomarkers for RA patients [6].

In the present study, we sought to see whether plasma IL-37 was elevated in RA patients and whether it was correlated with the disease activity. The relationship between IL-37 level and multiple Th1/Th2/Th17-related cytokine concentrations was also investigated.

## 2. Materials and Methods

### 2.1. Subjects and Clinical Assessment

This study was conducted in 34 RA patients who visited the Department of Rheumatology at Changhai Hospital between July 2011 and June 2012 and fulfilled the American College of Rheumatology (ACR) 1987 revised criteria for the classification of RA. Additional 34 age- and sex-matched healthy adults without any evidence of chronic inflammatory disease served as controls. This study was approved by the Ethics Committee of Changhai Hospital, and all study subjects signed written informed consent before participation in the study.

The baseline number of tender or swollen joints was calculated using the 28-joint disease activity score (DAS28); the erythrocyte sedimentation rate (ESR) was recorded; and the severity of pain reported by each patient was assessed using the pain visual analogue scale (VAS). Based on the DAS28 results, the 34 RA patients were subdivided into four groups: remission (DAS28 ≤ 2.6), mild (2.6 < DAS28 ≤ 3.2), moderate (3.2 < DAS28 ≤ 5.1), and severe (5.1 < DAS28). At the time of clinical assessment for disease activity, blood samples were collected for C-reactive protein (CRP) and cytokine level measurement.

### 2.2. Measurement of IL-37 Level

Plasma samples were collected intravenously, centrifuged (2,500 ×g for 10 min at 4°C), and stored at −20°C until analysis. The plasma concentration of IL-37 was measured using a commercial enzyme-linked immunosorbent assay (ELISA) kit (AdipoGen, San Diego, CA, USA).

### 2.3. Determination of Th1/Th2/Th17-Related Cytokine Concentrations

The following cytokines were measured simultaneously using a 17-plex test kit: TNF-*α*, IL-1*β*, IL-2, IL-4, IL-5, IL-6, IL-7, IL-8, IL-10, IL-12, IL-13, IL-17, G-CSF, GM-CSF, IFN-*γ*, MCP-1, and MIP-1*β*, broadly representative of Th1/Th2/Th17-related cytokines. These were measured using the Bio-Plex suspension array system (Bio-Rad Laboratories Inc., Hercules, CA, USA) that utilizes Luminex xMAP multiplex technology to enable simultaneous detection and quantitation of multiple different analytes in a single sample.

### 2.4. Statistical Analysis

Data analyses were performed with SPSS version 16.0. All descriptive variables were expressed as the mean (±SEM) or medium. Comparisons between two groups were performed using Student's *t*-test or nonparametric Mann-Whitney *U* test. The correlations between the concentration of each cytokine and DAS28 and CRP level were tested using Pearson's correlation test. For all tests, *P* values less than 0.05 were considered statistically significant.

## 3. Results

### 3.1. Clinical Characteristics of the Study Subjects

Recruited in this study were 34 Chinese RA patients (29 females and 5 males) with a median age of 56 years (range 33–82) and a mean duration of RA exceeding 10 years. At the time of recruitment, 19 patients were assessed as having active joint disease and the remaining 15 patients were in remission according to DAS28 scoring. The healthy controls had a median age of 53 years (range 28–76). There was no significant difference in age between the two groups (Table 1).

### 3.2. Correlation between IL-37 Level and Disease Activity

**Figure 1:**
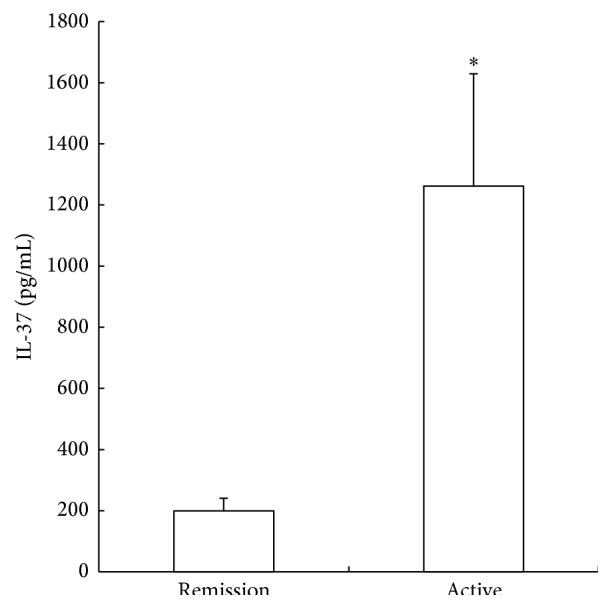
Plasma IL-37 levels in patients with rheumatoid arthritis. At the time of recruitment, 19 patients were assessed as having active joint disease and the remaining 15 patients were in remission according to DAS28 scoring. At the time of clinical assessment for disease activity, blood samples were collected and plasma IL-37 was determined using a commercial ELISA kit. Data are expressed as the mean ± SEM. ^*∗*^ *P* < 0.05 compared to the patients with RA in remission.

The plasma concentration of IL-37 was undetectable in the healthy controls (data not shown), while it was elevated markedly in the RA patients. Furthermore, IL-37 levels in patients with active RA were significantly higher than those in patients of remission (Figure 1). In addition, IL-37 levels were significantly correlated with DAS28 disease activity scores. However, no significant correlation between IL-37 level and CRP concentration was observed in our study (Table 2).

### 3.3. Correlations between IL-37 and Th1/Th2/Th17-Related Cytokines

Of the 17 Th1/Th2/Th17-related cytokines, TNF-*α*, IL-1*β*, IL-2, IL-4, IL-6, IL-7, IL-10, IL-12, GM-CSF, IFN-*γ*, and MCP-1 in the blood of RA patients were positively correlated with the DAS28 disease activity score (Table 3). The IL-37 level showed a significant correlation with IL-4, IL-7, IL-10, IL-12, and IL-13 levels in RA patients (Table 2).

## 4. Discussion

IL-37 has emerged as a natural suppressor of innate inflammatory and immune responses. It is considered a dual-function cytokine with intra- and extracellular properties [7]. It could suppress inflammation and be protective in several animal models, such as lipopolysaccharide-induced shock [3], concanavalin A-induced hepatitis [8], dextran sulfate sodium- (DSS-) induced colitis [9], hepatic ischemia/reperfusion (I/R) injury [10], renal I/R injury [11], cerebral I/R injury [12], obesity-induced inflammation and insulin resistance [13],* Aspergillus* infection [14], and experimental psoriasis [15]. It is reported to be elevated in patients with atopic dermatitis [16], chronic HBV infection [17], Guillain-Barre syndrome [18], acute coronary syndrome, Graves' disease [19], chronic inflammatory bowel disease [20], and systemic lupus erythematosus [21]. Meanwhile, decreased IL-37 expression was detected in patients with Vogt-Koyanagi-Harada disease [22], intervertebral disc degeneration [23], and Behçet's disease [24].

It was found in our study that plasma IL-37 level was elevated markedly in RA patients, while it was almost undetectable in the plasma of the healthy controls. In addition, increased IL-37 in the blood of the RA patients was positively correlated with the DAS28 disease activity score. Furthermore, IL-37 level was decreased in the RA patients of remission as compared with those in the patients with active RA. These findings are consistent with the data obtained from 50 RA patients in northeast China between 2011 and 2012 in a recent paper [25], which encourages us to hypothesize that IL-37 may be a potential biomarker for RA diagnosis, disease activity assessment, or curative effect observation.

Knowing that TH1/Th2/Th17-related cytokines play critical roles in the pathogenesis of RA, we detected the multiple cytokine concentration using a 17-plex test kit. It was found that, like TNF-*α*, IL-1*β*, IL-2, IL-4, IL-6, IL-7, IL-10, IL-12, GM-CSF, IFN-*γ*, and MCP-1, IL-37 in the blood of RA patients was also positively correlated with the DAS28 disease activity score, suggesting that both proinflammatory and anti-inflammatory cytokines are highly produced in RA patients, especially in those with active RA. As an anti-inflammatory cytokine, IL-37 could be upregulated by inflammatory stimuli and cytokines (TLR agonists, IL-1*β*, IL-18, TNF-*α*, and IFN-*γ*). Thus, it is reasonable to speculate that the proinflammatory cytokines in RA patients may stimulate IL-37 expression, and IL-37 may mediate a negative feedback mechanism to suppress excessive proinflammatory cytokines in RA patients. The elevation of anti-inflammatory cytokines including IL-37 may be an underlying mechanism to relieve joint inflammation and disease severity. However, these anti-inflammatory cytokines may still be too low to neutralize the deleterious effects of proinflammatory cytokines in progressive RA [26]. The uncontrolled inflammation might be due to the inadequate antagonism of anti-inflammatory cytokines against proinflammatory cytokines. This may be the reason why the anti-inflammatory cytokines including IL-37 only correlate with disease activity rather than with disease remission.

It was reported [3] that IL-37 expressing RAW cells produced higher IL-13 than mock ones upon LPS stimulation, and IL-37 transgenic mice produced higher IL-4 than wide type ones in response to LPS. IL-10 expression was greatly increased after IL-37 treatment in mice with aspergillosis [14]. IL-10 secretion was enhanced by LPS challenge in bone marrow-derived DCs from IL-37 transgenic mice other than wide type ones [27]. Serum IL-37 was positively correlated with IL-10 and negatively correlated with IL-12 in patients with tuberculosis [28]. Both IL-7 and IL-37 were elevated in patients with COPD compared with nonsmokers [29]. It was found in our study that IL-37 level exhibited a positive correlation with cytokines IL-4, IL-7, IL-10, IL-12, and IL-13. Yet we are not sure whether this is a cause-and-effect relationship (IL-37 stimulating these cytokines or vice versa) or an accompanying relationship (without any direct relationship between them; they just all elevate with the progression of the disease). To the best of our knowledge, the above cytokines are all involved in the progression of RA. IL-4 [30], IL-10 [31], and IL-13 [32] could relieve immunological bone destruction in RA patients. Both exacerbative [33] and suppressive [34] effects of IL-12 were reported in experimental arthritis. IL-7 is considered an important mediator in RA development [35]. Given the correlation between IL-37 and these cytokines, we suppose that IL-37 may also play an important role in RA, although experimental studies should be done in our future studies to examine the detailed molecular mechanisms and fully elucidate the regulatory network of IL-37 in RA. Moreover, the potential role of IL-37 as a biomarker and pharmacological target of RA also needs to be verified in larger sample studies.

In conclusion, the plasma concentration of IL-37 was elevated in patients with RA, especially in those with active RA. IL-37 level was correlated with the disease activity score, as well as IL-4, IL-7, IL-10, IL-12, and IL-13 levels in patients with RA. These findings suggest that IL-37 plays an important role in the pathogenesis of RA and may prove to be a potential biomarker of active RA.

## Figures and Tables

**Table 1:** Clinical characteristics of the study subjects.

Character	Medium (range)		
	Inactive RA patients (*n* = 15)	Active RA patients (*n* = 19)	Healthy controls (*n* = 34)
Age (yrs)	54 (33–82)	60 (35–78)	53 (28–76)
Gender (male/female)	4/11	1/18	6/28
Disease duration (yrs)	6 (0.25–30)	9 (0.5–27)	—
Swollen joint count	0 (0-0)	0 (0–20)	—
Tender joint count	0 (0-0)	0 (0–25)	—
Patient VAS	30 (10–60)	70 (30–80)	—
ESR (mm/h)	12 (2–21)	67 (7–110)	—
CRP (mg/L)	2.86 (1.07–18.80)	21.35 (1.85–47.10)	—
DAS28	2.16 (1.19–2.54)	5.75 (2.66–8.34)	—
Remission (DAS28 ≤ 2.6) (*n*)	15	—	—
Mild (2.6 < DAS28 ≤ 3.2) (*n*)	—	3	—
Moderate (3.2 < DAS28 ≤ 5.1) (*n*)	—	3	—
Severe (5.1 < DAS28) (*n*)	—	13	—

DAS28, disease activity score 28; VAS, visual analogue scale; NS, not significant.

**Table 2:** Correlations between IL-37 and DAS28, CRP, and Th1/Th2/Th17-related cytokines in patients with rheumatoid arthritis.

	Coefficient	*P*
DAS28	0.348^*∗*^	0.044
CRP	0.196	0.273
TNF-*α*	0.262	0.148
IL-1*β*	0.272	0.133
IL-2	0.290	0.108
IL-4	0.362^*∗*^	0.042
IL-5	0.310	0.085
IL-6	0.227	0.212
IL-7	0.526^*∗∗*^	0.002
IL-8	−0.028	0.878
IL-10	0.395^*∗*^	0.025
IL-12	0.451^*∗∗*^	0.010
IL-13	0.472^*∗∗*^	0.006
IL-17	0.096	0.600
G-CSF	0.319	0.075
GM-CSF	0.261	0.149
IFN-*γ*	0.328	0.067
MCP-1	0.321	0.073
MIP-1*β*	−0.269	0.137

Pearson's correlation test was used for statistical analysis. ^*∗*^ *P* value less than 0.05. ^*∗∗*^ *P* value less than 0.01.

**Table 3:** Correlations between plasma Th1/Th2/Th17-related cytokines and DAS28 in patients with rheumatoid arthritis.

	Coefficient	*P*
TNF-*α*	0.388^*∗*^	0.028
IL-1*β*	0.360^*∗*^	0.043
IL-2	0.398^*∗*^	0.024
IL-4	0.443^*∗*^	0.011
IL-5	0.243	0.180
IL-6	0.380^*∗*^	0.032
IL-7	0.365^*∗*^	0.040
IL-8	0.097	0.599
IL-10	0.439^*∗*^	0.012
IL-12	0.394^*∗*^	0.026
IL-13	0.297	0.099
IL-17	0.123	0.501
G-CSF	0.210	0.248
GM-CSF	0.403^*∗*^	0.022
IFN-*γ*	0.440^*∗*^	0.012
MCP-1	0.353^*∗*^	0.047
MIP-1*β*	−0.228	0.209

Pearson's correlation test was used for statistical analysis. ^*∗*^ *P* value less than 0.05.
